# Peri-Renal Lymphangiomatosis: A Case Report Presenting a Rare Disease at a Rarer Location

**DOI:** 10.7759/cureus.36766

**Published:** 2023-03-27

**Authors:** Muhammad Mehraiz Khan, Sravya R Mundla, Faisal Ehsan Cheema, Swaiyah Rehman, Tooba Anjum, Maggie James

**Affiliations:** 1 Radiology, Institute of Nuclear Medicine and Oncology Lahore (INMOL) Cancer Hospital, Lahore, PAK; 2 Internal Medicine, Apollo Institute of Medical Sciences and Research, Hyderabad, IND; 3 Diagnostic Radiology, Institute of Nuclear Medicine and Oncology Lahore (INMOL) Cancer Hospital, Lahore, PAK; 4 Internal Medicine, Federal Medical College, Islamabad, Islamabad, PAK; 5 Internal Medicine, Sri Ramaswamy Memorial (SRM) Medical College Hospital and Research Centre, Chennai, IND

**Keywords:** perinephric collections, benign tumor, peri-renal lymphangiomatosis, renal, lymphoangiomas

## Abstract

Renal lymphangiomatosis is a rare pathology wherein dilatation of perirenal, parapelvic, and intrarenal lymphatics is observed and can occur in both children and adults. It has no gender predilection and can present in unilateral and bilateral forms. Clinical symptomatology ranges from incidental findings to flank pain, hematuria, and abdominal swelling.

Radiological appearances may mimic renal cysts, peripelvic cysts, perinephric abscesses, or collections. This emphasizes the importance of developing familiarity with the imaging characteristics of this rare entity.

We present the case of an 11-year-old boy whose chief complaint was abdominal distension and bilateral flank pain. The radiological assessment revealed bilateral perinephric collections, which, along with clinical correlation, led to the diagnosis of bilateral peri-renal lymphangiomatosis.

## Introduction

Being a congenital benign lymphatic tumor, cystic lymphangiomatosis is most commonly seen during the first two decades of life. The majority of lymphangiomas (75%) occur in the neck region, whereas 20% of cases are seen in axillary regions [1]. The remaining 5% of the lesions are scattered in the body, primarily seen in the mediastinum, omentum, mesentery, retroperitoneum, pelvis, and colon [2]. However, renal incidence is extremely rare, and only a few cases have been documented in the literature so far. Clinical symptoms vary depending on the location. The malignant transformation of perirenal lymphangiomatosis is not reported in the literature. It carries a high misdiagnosis rate and can be confused with other renal cystic diseases, namely polycystic kidney disease, hydronephrosis, and para-pelvic cysts. However, imaging plays a vital role in identifying the condition and distinguishing it from its differentials.

## Case presentation

An 11-year-old male patient presented to the outpatient department of a local hospital with the complaint of bilateral flank pain associated with gradually progressive abdominal distension for three months. No complaints of fever, hematuria, or altered bowel habits were noted. There was no significant perinatal or family history. Medical and surgical histories were also unremarkable.

Physical examination revealed moderately appreciable swelling in bilateral flank regions, which appeared to be soft in consistency and fluctuant on palpation. Renal and liver function tests, complete blood counts, and serum electrolytes were within the normal range. The patient was advised to undergo a computed tomography scan for further evaluation.

**Figure 1:**
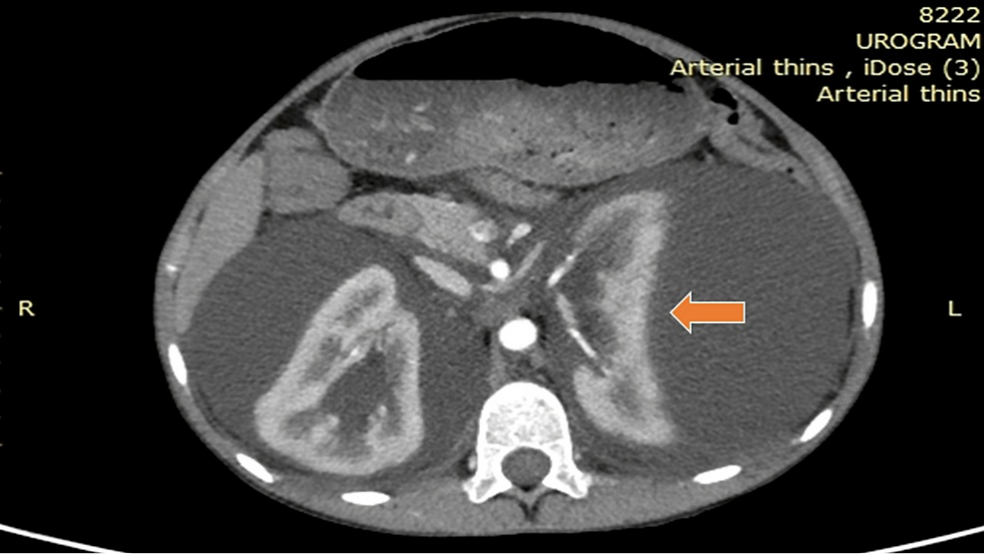
Axial view

**Figure 2:**
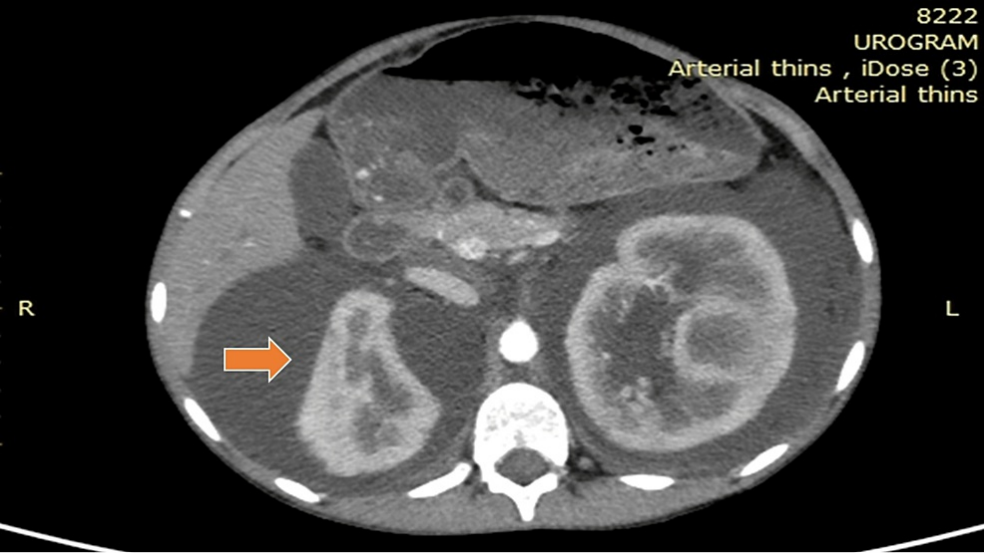
Axial view

**Figure 3:**
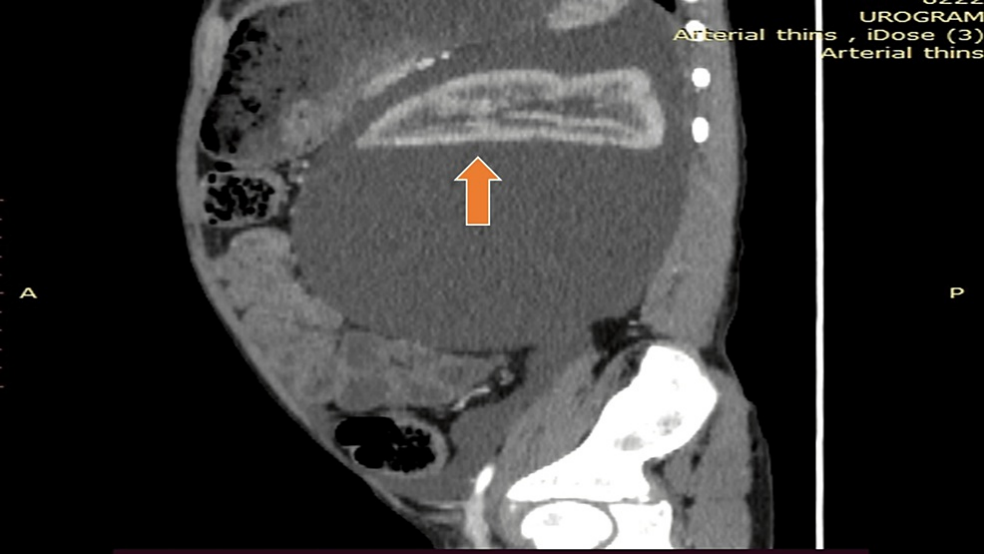
Sagittal view

On a CT urogram, non-enhancing abnormal fluid density and cystic collection with subtle septations were noted outlining the left kidney, measuring about 16.8 cm in maximum craniocaudal (CC) dimension. The left kidney appeared to be compressed and displaced anteriorly and medially. The left kidney otherwise was of normal size (craniocaudal length = 9.1 cm) with reduced parenchymal thickness secondary to extrinsic compression (Figures 1A, 2A, 3). No radio-opaque renal or ureteric calculus was noted. The left nephropyelogram showed no significant hydroureteronephrosis.

Similar fluid density cystic collection with few septations was also noted in the right perinephric region as well, measuring about 15 cm in maximum craniocaudal dimension. The right kidney appeared to be compressed and displaced medially. The right kidney otherwise was of normal size (craniocaudal length = 9.0 cm) with mildly reduced parenchymal thickness secondary to extrinsic compression (Figures 1B, 2B, 3). No radio-opaque renal or ureteric calculus was noted on the right side. The right nephropyelogram showed mild fullness of the collecting system secondary to extrinsic compression. Small ascites were noted. A minimal pericardial effusion was also seen. No pleural effusion was appreciated. No evident hepatic parenchymal focal lesion was seen. No evident para-aortic or pelvic lymphadenopathy was noted. CT findings were likely for peri-renal lymphangiomatosis.

Figures 1-2 show a contrast-enhanced CT of the abdomen (axial views): compressed and anteromedially displaced left kidney by a large peri-renal cystic collection (Figure 1); compressed and medially displaced right kidney by a large peri-renal cystic collection (Figure 2). Both kidneys, however, show normal perfusion.

**Figure 4:**
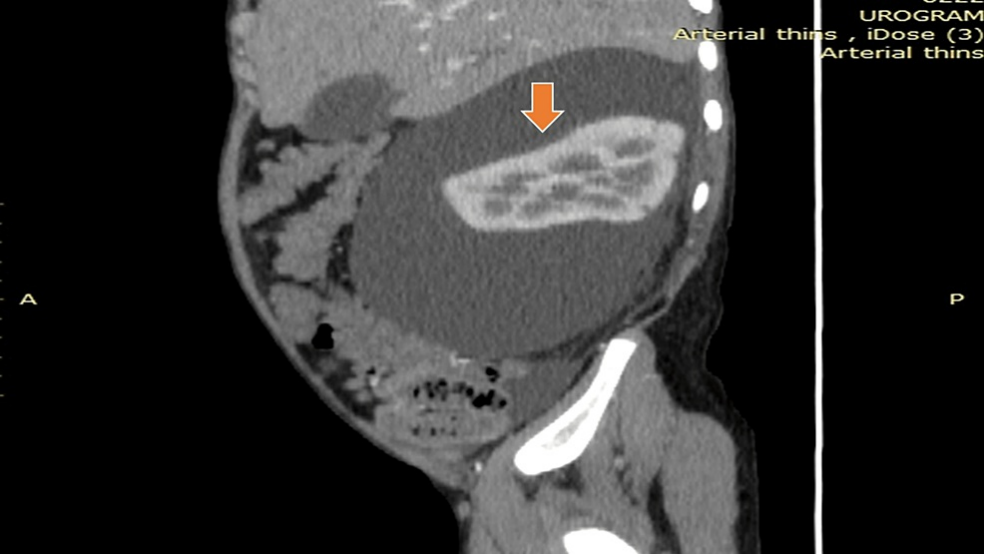
Sagittal view

Figures 3-4 show a contrast-enhanced CT of the abdomen (sagittal views). In Figures 3-4, the kidneys appear to be compressed by large peri-renal cystic collections with otherwise preserved renal perfusion bilaterally. Mild ascites are also noted.

**Figure 5:**
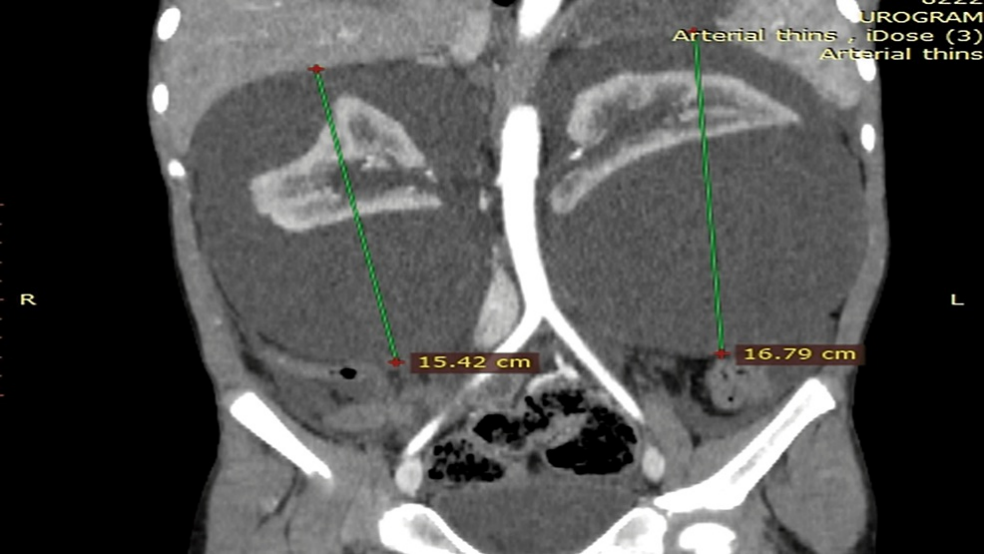
Coronal view

Figure 5 shows a contrast-enhanced CT of the abdomen (coronal view). Large fluid density collections are noted in bilateral peri-renal regions, measuring 15 cm on the right side and 16.8 cm on the left side in the maximum CC dimension. The collections are compressing and displacing both kidneys, which maintain normal perfusion.

**Figure 6:**
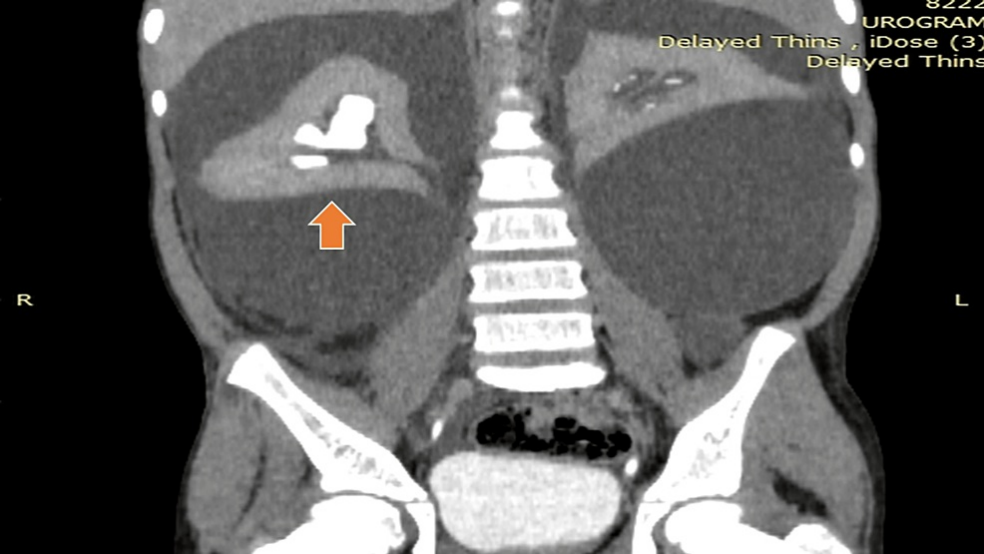
Delayed phase image

Figure 6 shows a contrast-enhanced CT of the abdomen (coronal view, delayed phase image). Mild fullness of the right pelvicalyceal system is noted secondary to extrinsic compression by a large peri-renal cystic collection. 

**Figure 7:**
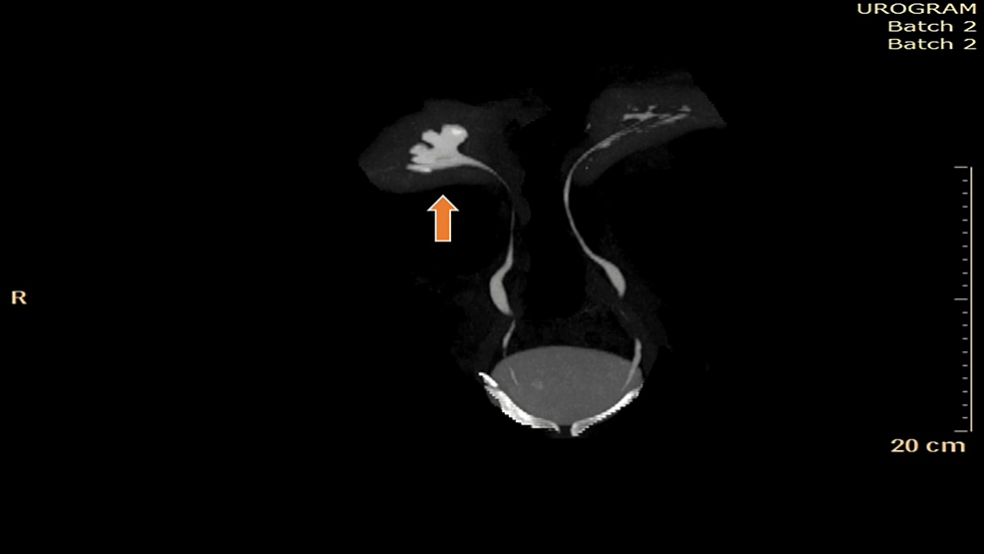
Maximum intensity projection image

Figure 7 shows the maximum intensity projection image of the bilateral renal collecting system. Mild fullness is noted in the right pelvicalyceal system secondary to the mass effect of large peri-renal cystic collection.

Following clinical and radiological correlation, a diagnosis of renal lymphangiomatosis was made. The patient was referred to the urology department for management and follow-up. The patient was managed conservatively owing to preserved renal function.

## Discussion

During embryonic life, abnormal proliferation and sequestration of lymphatic vessels leads to failure of establishment of normal communication with rest of the lymphatic channels. As a result, lymphatic fluid accumulates in the form of unilocular or multilocular cysts thus referred to as cystic lymphangiomas. Depending upon the size of lymphatic spaces, lymphangiomas have been classified into capillary and cavernous types. Though congenital, the lesion may manifest late in life owing to slow growth and localization in a clinically silent area of the body [3]. 

Peri-renal lymphangiomatosis can manifest at any age with no gender predilection. It can also involve one or both the kidneys. Symptomatology ranges from no clinical complaints to abdominal distension, flank pain, hematuria, lower extremity edema or hypertension. Renal failure may be seen in severe cases. Symptoms may even exacerbate in pregnant patients. Children mostly present with nephromegaly that may or may not be associated with obstructive uropathy [4]. 

Grajales et al. studied an unusual case of peripelvic lymphatic malformation presenting with gross hematuria and renal colic [5]. Ultrasonography and computed tomography are the main diagnostic imaging modalities. Imaging findings include thin-walled unilocular or multiloculated cysts or cysts with no discernable solid component. Intra-cystic contents vary from clear fluid to complicated fluid secondary to infection or hemorrhage within the cyst. Atypical features of cystic lymphangiomas can be better characterized by MR imaging. Owing to their cystic nature, lymphangiomas return low T1W and high T2W signals in the absence of any enhancing intra-cystic tissue. Hemorrhage can also be better identified on the T1W sequence [6-8].

Desai et al. reported the case of a six-year-old male patient with a large perirenal cyst that was found to be lymphatic in origin on imaging evaluation and underwent multiple surgical procedures for management [9].

Akand et al. reported a very rare case of left adrenal lymphangioma presenting as a cystic mass near the upper pole of the left kidney. The lesion was initially considered a renal cyst, but subsequent surgical excision and histopathological analysis revealed it to be a cystic lymphangioma of the left adrenal gland [10].

Differentials include polycystic kidney disease, renal lymphoma, urinoma, cystic renal dysplasia, nephroblastomatosis, and renal abscess. Clinical and imaging data are essential to developing a good diagnostic approach toward a definite diagnosis. However, in ambiguous cases, the definite diagnosis relies on histopathological findings [11].

Chua et al. described a rare case of renal lymphangioma seen in the form of a multi-cystic unilateral renal mass. They further documented the role of triparametric sonography (B-mode, Doppler, and contrast-enhanced ultrasonographic features) in the discrimination of renal lymphangiomas from other cystic renal lesions. Peri-pelvic or para-pelvic renal lymphangiomas usually mimic hydronephrosis. The diagnostic burden, therefore, lies on the shoulders of a competent radiologist as the clinical picture is non-specific [6].

Complications encountered in perirenal cystic lymphangiomas include intra-cystic infection, hemorrhage, cyst rupture, or renal hilar compression. The treatment plan is dependent on clinical manifestations, with asymptomatic lesions requiring no specific treatment except for follow-up imaging. A minimally invasive approach for typical cystic lymphangiomas consists of percutaneous aspiration of cyst contents with or without injection of a sclerosing agent. However, it carries a high risk of recurrence. In cases of overlapping diagnosis with a renal tumor, the lesion must be surgically excised to reduce the recurrence rate [12,13].

Uzzo et al. reported a 30-year-old male patient with right renal lymphangiectasia, severe and refractory hypertension, and end-organ damage. The patient was on five antihypertensives, but there was no significant improvement. However, after a detailed diagnostic workup and subsequent successful right laparoscopic nephrectomy, the patient experienced a significant regression in clinical symptoms [14].

## Conclusions

Perirenal lymphangioma is a rare benign tumor with compressive complications. Owing to typical imaging features, it is mainly diagnosed radiologically; however, some ambiguous cases may require histopathological correlation by needle biopsy or nephrectomy, whichever suits the clinical situation. Treatment strategies vary according to the clinical presentation of the disease. The condition, however, carries a good prognosis in the majority of cases.
